# Single-Cell Analysis of Fibroblast Subpopulations in Skin and Oral Mucosa

**DOI:** 10.1177/00220345251380210

**Published:** 2025-10-22

**Authors:** K. Prasongyuenyong, W.S. Kim, Z. Chen, K.I. Ko

**Affiliations:** 1 Department of Periodontics, School of Dental Medicine, University of Pennsylvania, Philadelphia, PA, USA; 2 Department of Oral and Maxillofacial Surgery, Faculty of Dentistry, Prince of Songkla University, Hatyai, Thailand

**Keywords:** wound healing, periodontal disease(s)/periodontitis, single cell RNAseq, inflammation, gingiva, connective tissue biology

## Abstract

Fibroblasts are the principal mesenchymal cell type found within the connective tissues of all organs. Once thought to play a passive role in tissue remodeling, fibroblasts have now emerged as a key player in regulating structural immunity and modulating the reparative injury response. A recent surge in single-cell RNA sequencing studies has advanced our understanding of the biology of fibroblasts, highlighting their cellular diversity and organization across health and diseased conditions at an unprecedented resolution. In this review, we discuss up-to-date literature on fibroblast subpopulations identified from 2 distinct barrier tissues: oral mucosa and skin. We focus on the transcriptomic signatures that distinguish subsets of fibroblasts in homeostasis and perturbed conditions (i.e., wound healing or chronic inflammatory diseases), and we link them to mechanistic studies that provide functional insights. A deeper understanding of fibroblast diversity and its functional significance may uncover tissue-specific roles in regeneration and immunomodulation, which will be crucial for the development of precision therapy that directly targets fibroblast subsets.

## Introduction

Fibroblasts were first described in the 19th century as spindle-shaped cells of the connective tissue. Perhaps owing to this designation based on morphology, there had been ambiguity to whether fibroblasts should be truly classified as a single-cell type. We now know that morphology alone is inadequate and does not convey the functional significance of fibroblasts. Fibroblasts are commonly identified by the positive cell surface protein expression of pan-fibroblast markers such as CD140A and/or CD90 and the lack of leukocytic, endothelial, epithelial, and erythrocyte lineage markers (CD45^-^, CD31^-^, CD326^-^, Ter119^-^, respectively). However, the aforementioned criterion is not an absolute definition of fibroblasts, since it also identifies multipotent skeletal/mesenchymal stromal cell subsets in the long bones (Ambrosi et al. 2019). Thus, single-cell RNA sequencing (scRNA-seq) studies on fibroblasts typically select for additional extracellular matrix genes, such as *Dcn*, *Col1a2*, *Col3a1*, and more. Besides their role in extracellular matrix deposition, fibroblasts influence the immune landscape during wound healing, cancer, and chronic inflammatory disorders (He et al. 2020; Correa-Gallegos et al. 2021). These diverse functions of fibroblasts across and within the organs implicate the existence of fibroblast subpopulations that can directly be linked to pathologic outcomes, which is less explored in the oral biology field.

The oral cavity is subject to repeated trauma from mastication and microbial insult. Despite this perpetual injury, oral mucosae heal exceptionally fast and with minimal scar formation (Iglesias-Bartolome et al. 2018). Moreover, the damage induced by periodontal pathogens in periodontitis is typically gradual and rarely results in sepsis. The resilient nature of the oral mucosa had been attributed to the neural crest developmental origin of oral fibroblasts (Rinkevich et al. 2015). In contrast, skin fibroblasts have multiple developmental origins: paraxial mesoderm gives rise to dorsal skin fibroblasts and lateral plate mesoderm to ventral trunk and limb skin, and, uniquely, facial skin fibroblasts are derived from the neural crest. Facial fibroblasts exhibit higher proliferative capacity and contribute to an improved wound-healing profile as compared with other skin regions (Usansky et al. 2021), which is linked to the continued expression of tissue-specific homeobox genes throughout adulthood (Pfeiferová et al. 2025). These studies link the developmental origin to the niche-specific function of the fibroblasts, but the distinguishing features between oral and skin fibroblasts at a single-cell level had been underexplored. Emerging scRNA-seq studies have addressed this challenge, revealing that multiple fibroblast subpopulations are found in the oral mucosa and skin and providing novel insights into understanding their functional significance in health and disease.

Oral and cutaneous barriers closely resemble each other at the histologic level. Yet, fibroblast composition and function in these anatomic sites are expected to differ. In this review, we provide an up-to-date review on research development in the single-cell biology of fibroblasts in the oral mucosa and skin. Specifically, we discuss the gene markers that define fibroblast diversity in these barrier tissues from mouse and human models. We further discuss the scRNA-seq and mechanistic studies that investigate the role of these fibroblast subpopulations in the context of wound healing and chronic inflammatory disease conditions.

## Overview of the Structural Similarities and Differences in Oral Mucosa and Skin

Oral mucosa and skin share structural similarities and differences (Fig. 1). In humans, keratinized oral mucosa, also known as gingiva, and skin possess stratified squamous epithelial structures. The underlying connective tissue compartments are divided into papillary and reticular layers based on location and extracellular matrix features. The papillary layer is located beneath the epithelium, in which thin collagen and elastin fibers form tightly packed projections into the epithelium. The reticular layer is deeper and possesses thick and interwoven collagen and elastic fibers, creating a meshwork that provides mechanical support. In palatal gingiva, there is a submucosal adipose layer that contains minor salivary glands, similar to a human subcutaneous adipose layer surrounding eccrine and apocrine glands. In contrast, skin has other specialized apparatuses, such as hair follicle, sweat glands, and arrector pili muscles that are not present on oral mucosal layers. In the human oral cavity, buccal mucosal lining consists of nonkeratinized epithelium and is highly elastic and rich in glandular tissues, contrasting gingiva that is immovable and keratinized. The dorsum of the tongue has a thick keratinized layer and specialized papillar structures, whereas the ventrum is thin, nonkeratinized, and highly vascular. The oral mucosae in mice share these features, although all regions of the murine oral mucosa are keratinized and the thickness of each histologic layer differs between species. This review focuses on fibroblasts that are found in the connective tissue layers of oral mucosa and skin.

**Figure 1.**
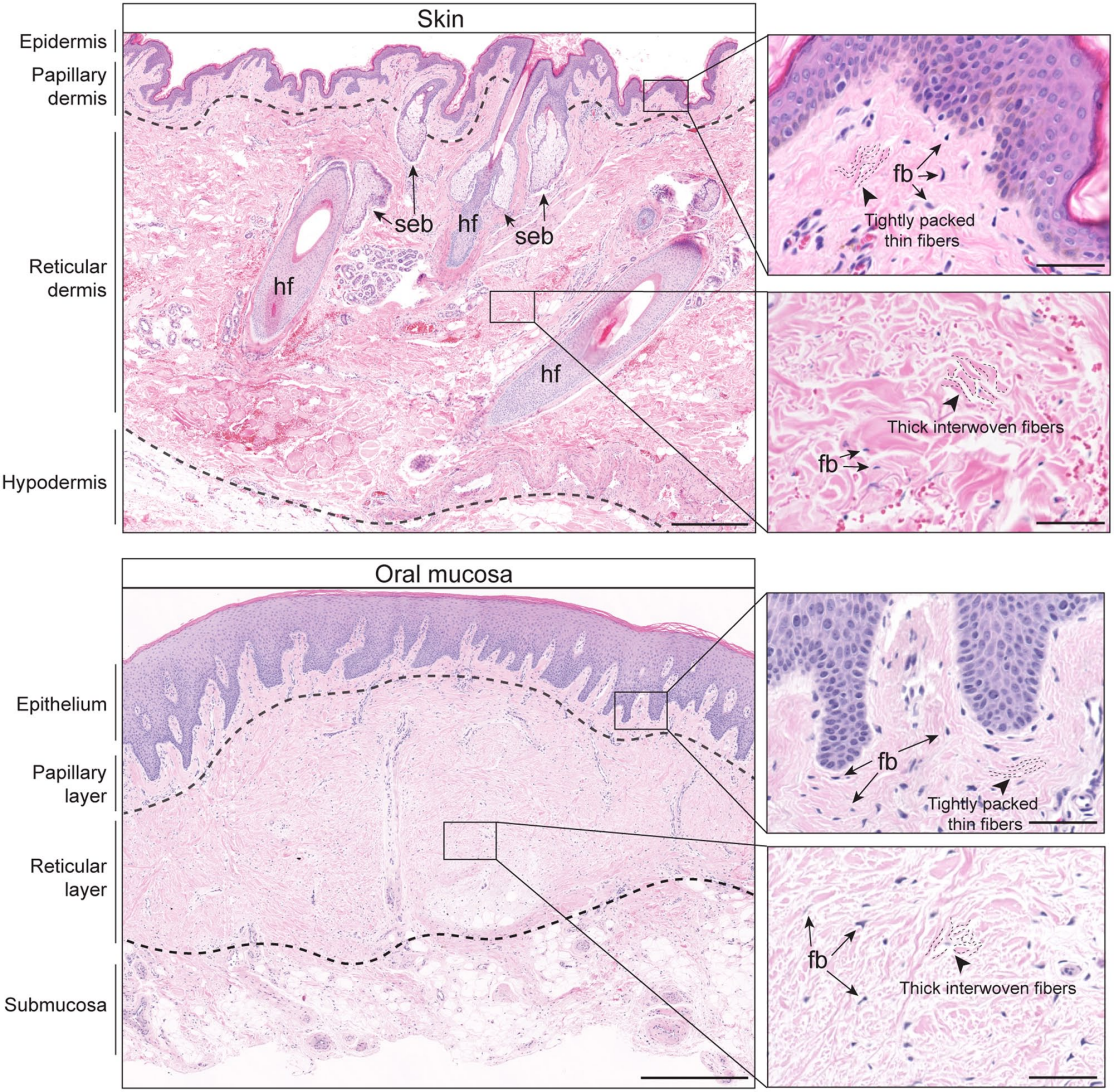
Histologic features of human skin and oral mucosa. Top: hematoxylin and eosin–stained image of human forearm skin shows different histologic compartments of epidermal, dermal, and hypodermal layers demarcated by dashed lines. Specialized appendages such as hair follicles (hf) and sebaceous gland (seb) are present in normal skin. Scale bar, 500 µm. Bottom: hematoxylin and eosin–stained image of human palatal gingiva with similarly layered architecture as compared with skin composed of keratinized stratified squamous epithelium, underlying lamina propria and submucosa. Scale bar, 1 mm. Inset images show high-magnification view of a papillary connective tissue layer that has thin and tightly packed collagen fibers and a reticular layer with thick and interwoven collagen fibers as indicated by dotted lines. Arrows point to spindle-shaped fibroblastic cells (fb). Scale bar, 50 µm.

## Fibroblast Heterogeneity in the Oral Mucosa and Skin during Homeostasis

### Dermal Fibroblasts

Since the 1970s, it was speculated that dermal fibroblasts from the papillary versus reticular layers were distinct by their differential proliferative capacity in vitro (Harper and Grove 1979). This was later demonstrated in murine models, in which fibroblasts within the papillary layer of neonatal skin were CD26^+^ (also known as DPP4) and the reticular layer and hypodermis consisted of DLK1^+^ or SCA1^+^ fibroblasts, respectively (Driskell et al. 2013). These early findings have been validated and utilized by multiple scRNA-seq studies (Thompson et al. 2022; Almet et al. 2025). Joost et al. (2020) identified 4 distinct fibroblast subpopulations from a total of 340 cells, each marked by the differential expression of *Dcn*, *Gpx3*, *Sparc*, or *Plac8* in adult murine skin. *Sparc^+^* and *Dcn^+^* fibroblasts were found in the papillary and reticular layers and were predicted to play key roles in extracellular matrix and cytoskeleton organization. In contrast, *Plac8^+^* and *Gpx3^+^* fibroblasts were located in the adventitial and hypodermal layers, respectively, which were validated in situ. Almet et al. (2025) recently integrated fibroblast datasets from the murine scRNA-seq studies that examined unwounded and wounded conditions, which included a total of 107,778 cells. In unwounded conditions (Haensel et al. 2020; Phan et al. 2020), the fibroblast clusters with gene expression scores corresponding to upper dermis separated distinctly from those in lower dermis. *Dpp4*, *Crabp1*, *Fabp1*, and *Prdm1* were enriched in subcluster fibroblast VIII (upper dermis), whereas *Ly6a*, *Dlk1*, and *Cnn1* were found in fibroblast III (lower dermis; Almet et al. 2025), thereby displaying a distinct spatial organization within skin.

In an scRNA-seq study, Tabib et al. (2018) found 2 major dermal fibroblast populations in healthy human forearm skin, in which *DPP4^+^*/*SFRP2^+^* fibroblasts had enriched gene expression for matrix deposition whereas *FMO1^+^*/*LSP1^+^* fibroblasts largely expressed the *CXCL12* chemokine. In a study by Boothby et al. (2021), *DPP4* and *SFRP2* were consistently detected in nonlesional human skin fibroblasts, although the focus was on fascial fibroblasts. Another study identified 5 fibroblast clusters in normal human abdominal skin from which *DPP4*, *CD74*, and *COL6A5* emerged as key markers in distinguishing subpopulations (Philippeos et al. 2018). This study demonstrated a spatial localization of COL6A5^+^ fibroblasts to the papillary dermis, whereas DPP4 expression was more diffuse throughout reticular dermis, as supported by a study that examined trunk skin samples (Vorstandlechner et al. 2020). Similarly, an scRNA-seq analysis of inguinoiliac skin identified 4 fibroblast meta-clusters (Solé-Boldo et al. 2020): *APCDD1^+^* papillary fibroblasts, *CTHRC1^+^* reticular fibroblasts with a secretory phenotype, *CCL19^+^APOE^+^* inflammatory fibroblasts, and a mesenchymal population associated with dermal papilla niche. To resolve the intrinsic heterogeneity in scRNA-seq results, Ascensión et al. (2021) integrated human skin datasets from 4 published studies and identified 3 major dermal fibroblast subpopulations. These included fibroblasts enriched in extracellular matrix–related genes such as *ELN*, *MMP2*, and *SFRP2*; those that express inflammation-related genes such as *APOE*, *C7*, and *CYGB*; and those with genes implicated in specialized dermal functions such as *DKK3*, *TNN*, and *TNMD*. Together, these complementary scRNA-seq findings identify gene features and a spatial niche for each fibroblast subpopulation, as summarized in Figure 2 and Table 1.

**Table 1.** Single-Cell RNA Sequencing Studies of Fibroblasts in Homeostatic Skin and Oral Mucosa.

Reference: Species and Tissue Type	Fibroblast Subsets Reported	Inferred Function/Spatial Information	Experimental Validation: Database Identifier
**Skin database**			
Joost et al. (2020) Mouse Dorsal skin	FIB1: *Col1a1^+^*, *Sparc^+^* FIB2: *Dcn^+^*, *Lum^+^* FIB3: *Cxcl12^+^*, *Gpx3^+^* FIB4: *Mfap5^+^*, *Plac8^+^*	FIB1: collagen production, vesicular trafficking and energy metabolism FIB2: extracellular matrix production, migration and cytoskeletal remodeling FIB3, 4: not specified	Fluorescent in situ hybridization for *Sparc^+^* (FIB1), *Dcn^+^* (FIB2), *Gpx3^+^* (FIB3), and *Plac8^+^* (FIB4) fibroblasts • GSE129218
Haensel et al. (2020) Mouse Dorsal skin	Fibroblast I: *Col1a1^+^*, *Col1a2^+^* Fibroblast II: *Cpz^+^*, *Ctsk^+^*, *Col1a2^+^* Fibroblast III: *Aqp1^+^*, *Ifitm1^+^*, *Prom1^+^*, *Crabp1^+^* Myofibroblast: *Acta2^+^*, *Tagln^+^*	Fibroblast I, II, III, and myofibroblast: not specified	NA • GSE142471
Phan et al. (2020) Mouse Dorsal skin	Papillary: *Dpp4^+^*, *Dlk1^-^*, *Ly6a^-^* Reticular: *Dpp4^-^*, *Dlk^+^*, *Ly6a^+/-^* Hypodermal: *Dpp4^-^*, *Dlk1^-^*, *Ly6a^+^* Fascia: *Gpx3^+^* Dermal sheath: *Acan^+^*, *Acta2^+^* Dermal papilla: *Corin^+^*	Papillary, reticular, fascia: not specified Hypodermal: maintenance of lower skin Dermal sheath, dermal papilla: hair follicle development and maintenance	Immunofluorescence confirmed expression of *Dpp4* in papillary layer, *Dlk1* and *Pref1* for reticular and hypodermis, and *Sca1* for hypodermis • GSE153596
Thompson et al. (2022) Mouse Dorsal skin	Papillary: *Dpp4^+^* Dermal papilla: *Corin^+^* Reticular: *Ly6a^+^* Fascia: *Plac^+^* Arrector pilli: *Dlk1^+^*, *Pref1^+^* Preadipo: *Dlk1^+^*, *Pref1^+^*	NA	NA • GSE189210
Almet et al. (2025) Mouse Mixed	FIB-I: *Crabp1^+^*, *Col7a1^+^* FIB-II: *Col14a1^+^*, *Mgp^+^* FIB-III: *Pcolce2^+^*, *Ndufa4/2^+^* FIB-IV: *Plac8^+^*, *Ptx3*, *Prg4^+^* FIB-V: *Tyrobp^+^*, *Lyz2^+^* FIB-VI: *Col5a3^+^*, *Prss23^+^* FIB-VII: *Igfbp2^+^*, *Megf6^+^*, *Col25a1^+^* DP: *Igfbp3^+^*, *Bmp4^+^* FIB-VIII: *Coch^+^*, *Dkk2^+^* FIB-IX: *Lgals7^+^*, *S100a14^+^* FIBX: *Nr2f2^+^*, *Cldn1^+^*	FIB-I, V: found in upper dermis in early stages of large wounds FIB-II, VI: found in lower dermis in early stages of large wounds with immunomodulatory function FIB-III: found in unwounded lower dermis FIB-IV, IX, X: found in lower dermis in early stages of small wounds FIB-VII: found in upper dermis in late stages of large wounds FIB-DP, VIII: found in upper dermis of unwounded skin	Fluorescent in situ hybridization for *Prg4^+^*, *Col25a1^+^*, and *Pamr1^+^* fibroblasts • GSE153596 • GSE142721 • GSE113854 • GSE108677 • GSE141814
Boothby et al. (2021) Human Healthy and eosinophilic fasciitis skin	fFB1: *DPP4*^+^, *POSTN*^+^, *SFRP2*^+^, fFB2: *ITM2A*^+^, *CHRDL1*^+^, *TIMP3*^+^, FB1: *ANXA1*^+^, *PLAT*^+^, FB2: *WNT2*^+^, FB3: *DPP4*^+^, *CTHRC1*^+^, *ACKR3*^+^, FB4: *CSRP2*^+^, *HEG1*^+^, FB5: *TMEM100*^+^, *OGN*^+^, FB6: *FRSS23*^+^, *LAMC1*^+^, FB7: *ANXA3*^+^, FB8: *SFRP4*^+^, *FN1*^+^	fFB1: similar to TIFF and Thbs4^+^ in murine fFB2: similar to TIFF and Thbs4^+^ in murine FB1 to 8: not specified	NA • GSE183031
Boothby et al. (2021) Mouse Healthy and T-reg depletion skin from dorsal skin	TIFF: *Sfrp2^+^*, *Dpp4^+^*, *Pi16^+^*, *Il13ra1^+^* Thbs4^+^ FB: *Clip^+^*, *Thbs4^+^* Apoe^hi^ FB: *Apoe^+^*, *Apod^+^* Preadipocyte: *Pparg^+^*, *Fabp4^+^* Coch^+^ FB: *Coch^+^* Mup20^+^ FB: *Mup20^+^* Coll^hi^ FB: *Col16a1^+^*, *Col1a2^+^* Grem1^+^ FB: *Grem1^+^*, *Igfbp2^+^* Mural1: *Stmn2^+^*, *Wif1^+^* Mural2: NA Mural prolif: *Cdc20^+^*, *Ccna2^+^*, *Mki67^+^*	TIFF: stromal-Th2 interaction, found in subdermal layer, similar to fFB1, 2 in human Thbs4^+^: similar to fFB1, 2 in human Apoe^hi^ FB, preadipocyte, Coch^+^ FB, Mup20^+^ FB, Coll^hi^ FB, Grem1^+^ FB, Mural1, Mural2, Mural prolif: not specified	Immunofluorescence staining and flow cytometry for *Dpp4^+^*(*Cd26*) fibroblasts • GSE183031
Tabib et al. (2018) Human Dorsal midforearm	Cluster 0: *SFRP2/DPP4^+^*, *WIF1/NKD2^+^* Cluster 1: *FMO1^+^*, *LSP1^+^*, *AADAC^+^*, *RAMP2^+^* Cluster 2: *SFRP2/DPP4^+^*, *FBLN1^+^* *C1R^+^* Cluster 3: *IGFB5^+^* Cluster 4A: *CRABP1^+^*, *TNN^+^* Cluster 4B: *COL11A1^+^*, *DPEP1^+^* Cluster 5: *FMO1^+^*, *LSP1^+^*, *CXCL12^+^*, *C7^+^* Cluster 6: *SFRP2/DPP4^+^*, *PRG4^+^*, *LINC01133^+^* Cluster 7: *ANGPTL7^+^*, *C2orf40^+^*	Cluster 0, 2, 3, 6: promotes fibrosis Cluster 1: negative regulation of cell migration	Immunofluorescence staining for colocalization of *SFRP2^+^* and *DPP4^+^* as well as *FMO1^+^* and *LSP1^+^* • https://dom.pitt.edu/wp-content/uploads/2018/10/Skin_6Control_rawUMI.zip
Philippeos et al. (2018) Human Abdominal skin	Group 1: *CD26^+^* Group 2: *RGS5^+^* Group 3: *COL6A5^+^*, *COL23A1^+^*, *HSPB3^+^* Group 4: *MFAP5^+^*, *PRG4^+^*, *CD26^+^* Group 5: *CD74^+^*, *CLDN5^+^*	Group 1: not specified Group 2: mixed group that contains cells with alternative identity Group 3: papillary fibroblasts Group 4: reticular fibroblasts Group 5: mixed group that contains preadipocytes and TIE2-monocytes/macrophages	Immunofluorescence staining for *COL6A5^+^* (group 3), *CD26^+^* (group 1), *MFAP5^+^* (group 4), and *RGS5^+^* (group 2) fibroblasts • GSE109822
Vorstandlechner et al. (2020) Human Trunk skin	Fb1: *MFAP5^+^*, *FAP^+^*, *DPP4* ^+^ Fb2: *THY1^+^*, *APOE^+^* Fb3: *APCDD1^+^* Fb4: *B4GALT1^+^* Fb5: *CXCL1^+^* Fb6: *WIF1^+^*	Fb1: extracellular matrix assembly and remodeling Fb2, 5: immune response and leukocyte migration Fb3: cartilage development Fb4: growth factor signaling Fb6: IFN-γ and NF-κB signaling	Immunofluorescence staining for *DPP4^+^* fibroblasts • Available upon request
Solé-Boldo L et al (2020) Human Sun-protected skin	Mesenchymal: *CTHRC1^+^* Proinflammatory: *CCL19^+^* *APOE^+^* Secretory/papillary: *APCDD1^+^* Secretory/reticular: *ASPN^+^*	Mesenchymal: structural and extracellular matrix organization in reticular layer Proinflammatory: cell chemotaxis and response to wound healing Secretory/papillary: structural and extracellular matrix organization in papillary layer Secretory/reticular: extracellular matrix organization	Fluorescent in situ hybridization for secretory fibroblasts; *CTHRC1^+^*, *APCCD1^+^*, proinflammatory fibroblasts; *CCL19* and *APOE* and mesenchymal fibroblasts; *ASPN* • GSE130973
Ascensión et al. (2021) Human Mixed	A: *ELN^+^*, *MPP2^+^*, *QPCT^+^*, *SFRP2^+^* B: *APOE^+^*, *C7^+^*, *CYGB^+^*, *IGFBP7^+^* C: *DKK3^+^*, *TNMD^+^*, *TNN^+^*, *SFRP1^+^*	A: extracellular matrix homeostasis B: promotes inflammation C: function in dermal papilla/dermohypodermal junction	Immunofluorescence staining for type A (*SFRP2^+^*), type B (*CXCL12^+^*), and type C (*SFRP1^+^*) fibroblasts • GSE130973 • GSE147424
Vorstandlechner et al. (2021) Human Healthy skin and scar from abdominal skin	FB 1-7: differential markers not specified	FB1-3: *DPP4*, *PLAU* essential for TGF-β1–mediated myofibroblast development	Immunofluorescence staining for *DPP4^+^* and *PLAU^+^* fibroblasts • GSE156326
**Oral database**			
Ko et al. (2023) Mouse Hard palate	Cluster 1: *Sca1^+^* Cluster 2: *Pi16^+^* Cluster 3: *Ecrg4^+^* Cluster 4: *Prrx1^+^/Wif1^+^* Cluster 5: *Grem1^+^* Cluster 6: *Klf5^+^*	Cluster 1: inflammatory function Cluster 2: TGFB signaling Cluster 3: extracellular matrix formation Cluster 4: WNT signaling Cluster 5, 6: not specified	Immunofluorescence staining showed *Pi16^+^* and *Sca1^+^* staining in reticular and submucosal lamina propria, respectively • GSE217720
Overmiller et al. (2024) Mouse Skin and buccal mucosa	Dermal fibroblast 1: *Col1a1^+^*, *Lum^+^*, *Tgfbi^+^* Dermal fibroblast 2: *Spon2^+^*, *Cxcl1^+^*, *Tshz3^+^* Stromal fibroblast 1: *Cxcl1^+^*, *Cxcl2^+^*, *Plac8^+^* Stromal fibroblast 2: *Col6a5^+^* Dermal papilla: *Bcl2^+^*, *Dkk2^+^* Dermal sheath: *Mylk^+^*, *Tshz3^+^*, *Slit2^+^*	Dermal fibroblast 1: extracellular matrix production Dermal fibroblast 2: inflammatory function Stromal fibroblast 1, 2: inflammatory and associated with hair follicle Dermal papilla and sheath: maturation of dermal papilla and maintenance of hair follicle cycle	NA • GSE280088
Williams et al. (2021) Human Tooth-associated gingiva and buccal mucosa	H.Fib 1.1: *SPARCL1^+^*, *PTGDS^+^*, *PLAC9^+^* H.Fib 1.2: *CXCL13^+^*, *CTHRC1^+^*, *POSTN^+^* H.Fib 1.3: *APCDD1^+^*, *DOI2^+^*, *IGFBP2^+^* H.Fib 1.4: *CCL19^+^*, *APOE^+^*, *CYR61^+^* H.Fib 1.5: *CFD^+^*, *GSN^+^*, *APOD^+^*	H.Fib 1.1: initiation of translation H.Fib 1.2: tissue remodeling H.Fib 1.3: regulation of leukocyte proliferation H.Fib 1.4: granulocyte chemotaxis H.Fib 1.5: activation of complement cascade	NA • GSE164241
Caetano et al. (2021) Human Healthy and Inflamed gingiva, periodontitis	S0: *PDGFRA^+^*, *WNT5A^+^*, *IGF1^+^*, *POSTN^+^* S2: *GREM1^+^*, *SERP1^+^*, *APCDD1^+^*, *DKK3^+^* S4: *OSR2^+^*, *FGFR1^+^*, *SOX4^+^*, *TBX3^+^* S5: *ILR1^+^*, *IFNGR1^+^*, *TNFRS11B^+^* S6: *CD74^+^*, *AREG^+^*	S0: tissue repair S2: negative regulation of WNT S4: collagen IV, development S5: IFN-γ signaling and osteoclast activity S6: IFN-γ signaling and T-cell activation	NA • GSE152042
Caetano et al. (2023) Human Healthy and inflamed gingiva, periodontitis	Fb1: reticular FB Fb2: *GREM1^+^*, *SFRP1^+^* Fb3: reticular FB Fb4: immune associated Fb5: *RAC2^+^*, *LCP1^+^*, *TNFRS11B^+^*	Fb2: epithelial maintenance through *RSPO1*, *CLEC11A*, and *FLRT2* Fb5: cytokine production (*CXCL8*, *CXCL10*) Fb1, 3, 4: not specified	Immunostaining validated Fb5 to be localized in B cell–enriched inflammatory region • GSE206621
Ko et al. (2023) Human Hard palate	Fibroblast 1: *PRRX1*^hi^ Fibroblast 2: not specified Fibroblast 3: *CXCL1^+^*, *CCL2^+^* Fibroblast 4 to 7: not specified	Fibroblast 1: WNT signaling Fibroblast 3: inflammatory phenotype Fibroblast 2, 4 to 7: not specified	Immunofluorescence staining for fibroblasts from multiple oral gingival tissues • GSE217720
Zhao et al. (2022) Human Buccal mucosa	Fibroblasts: *DCN^+^*, *COL1A2^+^* Myofibroblasts: *ACTA2^+^*	Fibroblasts: express *CXCL1*, *CCL2*, and *CXCL12* and may interact with leukocytes Myofibroblast: not specified	NA • Available upon request
Zhou et al. (2025) Human Tongue from healthy and chronic hyperplastic candidiasis	Analysis of fibroblast subsets not reported	Immune cell trafficking	NA • PRJNA1141396

NA, not available.

**Figure 2.**
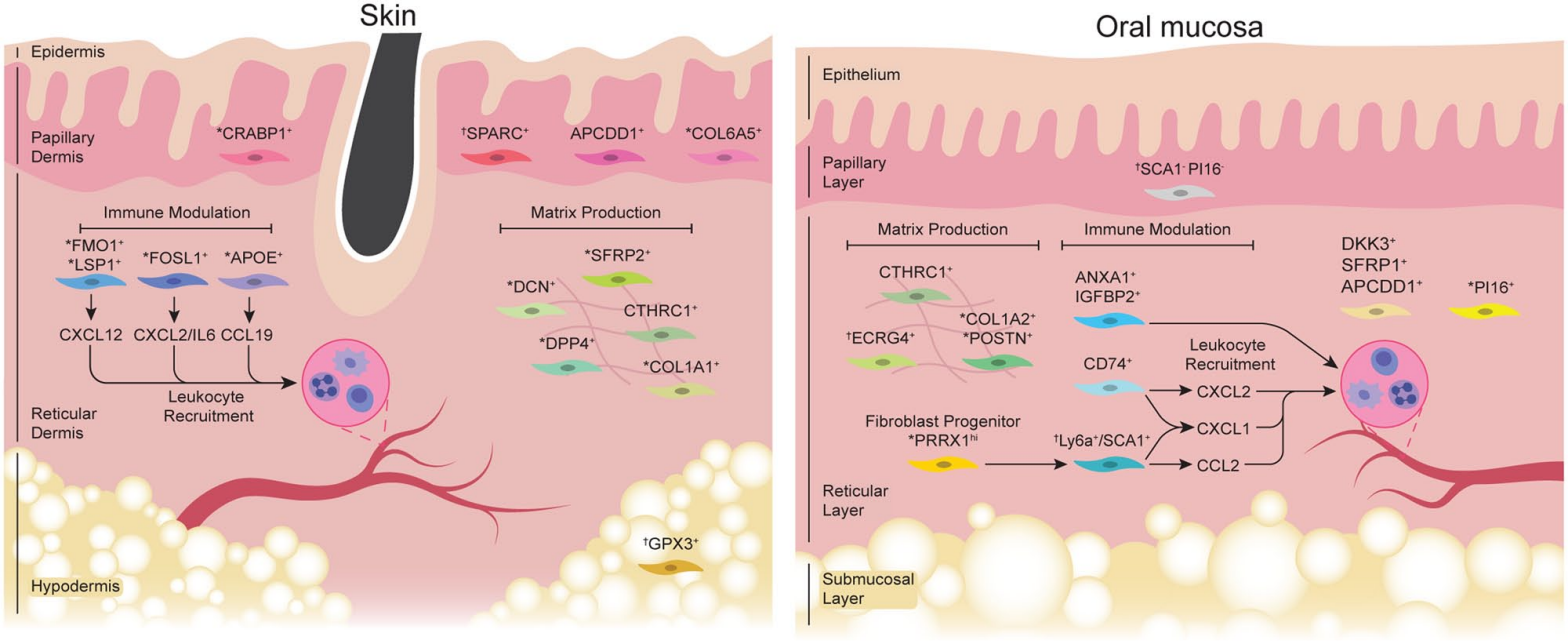
Fibroblast subpopulations in skin and oral mucosa during homeostasis. Left: fibroblast subpopulations identified from normal skin. The papillary layer is composed of *APCDD1^^+^^, CRABP1^+^, SPARC^^+^^*, and *COL6A5^^+^^* fibroblast subsets. The reticular layer contains matrix-secreting fibroblasts, including *CTHRC1^+^, COL1A1^+^, DPP4^+^, DCN^+^,* and *SFRP2^+^*, and immunomodulatory fibroblasts characterized by elevated expression of *APOE* and *FMO1/LSP1*, which also express *CCL19* and *CXCL12* for regulating leukocyte recruitment. *GPX3*^+^ fibroblasts are found in the hypodermis. Right: oral fibroblast subsets identified during homeostasis. When compared with skin, papillary oral fibroblasts remain undercharacterized. The reticular layer is composed of matrix-producing fibroblasts, including *COL1A2^+^/POSTN^+^, CTHRC1^+^*, and *ECRG4*^+^ fibroblasts. Immunomodulatory fibroblasts in the reticular layer, such as *CD74*^+^ and *ANXA1/IGFBP2*^+^ fibroblasts, express *CXCL1* and *CXCL2* for neutrophil recruitment. In anterior palatal gingiva, *PRRX1*^hi^ fibroblast progenitors give rise to *SCA1*^+^ submucosal fibroblasts that express chemokines. Other oral fibroblast populations, such as *PI16^+^, DKK3^+^, SFRP1^+^, and/or APCDD1^+^* fibroblasts, remain functionally uncharacterized. Human fibroblast subsets are illustrated unless indicated as follows: ^*^Fibroblast subset reported from human and murine models. ^†^Fibroblast subset reported in murine models only.

### Oral Fibroblasts

Our group reported that murine palatal gingiva harbors 6 fibroblast subtypes as identified from the scRNA-seq (Ko et al. 2023). The top 4 fibroblast clusters had enriched expression of *Ly6a* (encoding for SCA1), *Pi16*, *Ecrg4*, and *Wif1*. Validation experiments revealed that *Ly6a^+^*/SCA1^+^ fibroblasts were found in the submucosal layer and had enriched expression of *Ccl2*, *Ccl7*, and *Cxcl1*, whereas *Pi16^+^* fibroblasts resided in the reticular layer. The study further characterized a *Prrx1*^hi^/*Wif1^+^* subset as a fibroblast progenitor population that differentiates into and replenishes *SCA1*^^+^^ and *PI16*^^+^^ fibroblasts under homeostatic or wound-healing conditions. A recent scRNA-seq study directly compared murine dorsal skin and buccal mucosa (Overmiller et al. 2024) and found a relative absence of *Dpp4^+^* stromal cluster 2 in the buccal mucosa as compared with skin. This finding is consistent with the reports that link *DPP4*^+^ fibroblasts to skin fibrosis (Vorstandlechner et al. 2021).

There is an abundance of human scRNA-seq studies that examine healthy oral tissues. Anterior hard palate contains *PRRX1*^hi+^ fibroblasts with a pseudotime trajectory toward a fibroblast cluster that expresses *CXCL1* and *CCL2* (Ko et al. 2023). This *PRRX1*^hi^ fibroblast subset was enriched with Wnt-modulatory genes such as *WIF1*, *SFRP2*, and *WNT16*. Similarly, Caetano et al. (2021) found that tooth-associated healthy gingiva has fibroblast clusters that express genes for matrix production and tissue repair (*COL1A2*, *POSTN*), the Wnt-signaling pathway (*SFRP1*, *APCDD1*, *DKK3*), and immune modulation (*CXCL1*, *CXCL2*, *CD74*, *HLA-DR*). In a follow-up study, the authors spatially mapped *SFRP1+*/*POSTN+* fibroblasts to the subepithelial niche with a proposed function in epithelial maintenance, whereas the fibroblasts enriched in cytokine transcripts were found near the sulcular pocket niche (Caetano et al. 2023). Williams et al. (2021) demonstrated that human buccal mucosae exhibit different proportions of fibroblast subpopulations when compared with the marginal gingiva from clinically healthy patients. Specifically, fibroblast subtype 1.3 was more abundant in the buccal mucosa and had enriched expression of *APCDD1*, *WNT5A*, and *DKK3*, whereas marginal gingiva had an increased proportion of fibroblast type 1.2, which had elevated expression of *CXCL13*, *CTHRC1*, and *POSTN*. Buccal mucosa and gingiva had fibroblast types 1.3, 1.4, and 1.5, which were implicated in biological processes that promote leukocyte proliferation (*ANXA1*, *IGFBP2*), granulocyte chemotaxis (*CXCL1*, *2*, *8*), and complement activation (*C3*, *CFD*; Williams et al. 2021). These results agree with the study by Zhao et al. (2022) reporting that fibroblasts from buccal mucosae express *CXCL1*, *CCL2*, and *CXCL12* and likely communicate with *CXCR3^+^*, *ACKR1^+^*, and *CCR2^+^* plasmacytoid dendritic cells and T cells. Fibroblasts in the healthy tongue also express CXCL- and CCL- chemokines and likely communicate with immunocytes, although subset-specific analysis was not reported (Zhou et al. 2025). Together, these studies suggest that active leukocyte recruitment is an important aspect of normal mucosal immune surveillance during homeostasis and may be driven by distinct oral fibroblast subsets (Fig. 2).

## Fibroblast Subpopulations in Wound Healing

Wound healing is a complex biological process characterized by hemostasis, inflammation, proliferation, and remodeling. Fibroblasts play a critical role in every phase, with distinct subpopulations emerging over time and across spatial niches in dermis (Fig. 3A). In a multi-omics study, Foster et al. (2021) examined murine dorsal skin wound healing across 2-, 7-, and 14-d time points and found an “activated-responder” fibroblast subset that expressed inflammation-related genes such as *Ccl6*, *Il1b*, and *Il1a*. The study also demonstrated an emergence of mechanofibrotic fibroblasts that expressed *Dpp4*, *Fn1*, *Acta2*, and *En1* during the proliferative phase. Almet et al. (2025) integrated public scRNA-seq datasets from 5 independent studies that examined skin wound healing in mice. They reported that *Ly6a^+^* fibroblasts predominate the lower dermis by day 2 and persist throughout wound closure time point. By comparison, *Crabp1^+^* fibroblasts were located in the upper dermis and emerged by day 7 postwounding, with a presumed function in wound remodeling as *Crabp1* is associated with regenerative competence (Abbasi et al. 2020; Phan et al. 2021). The study also identified proinflammatory *Prg4^+^* fibroblasts that appear transiently in the early stages of healing and are later replaced with *Col25a1^+^* and *Pamr1^+^* fibroblasts that occupy the upper and lower regions of the scar, respectively.

**Figure 3.**
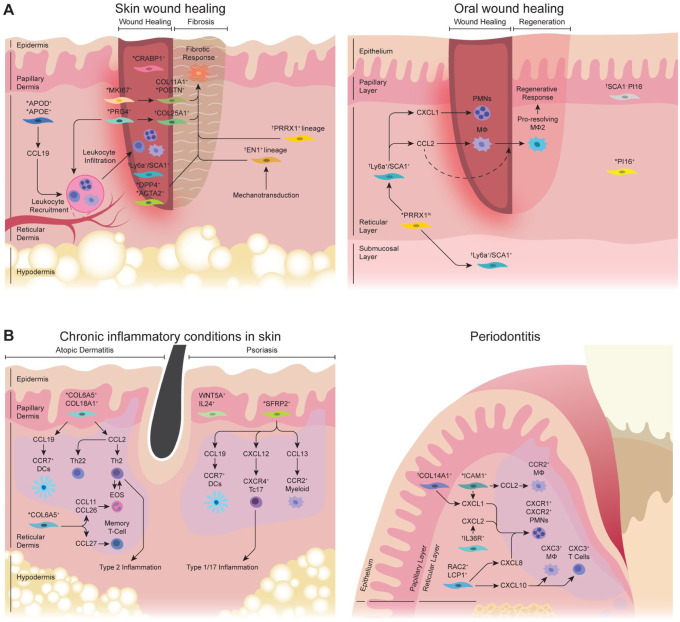
Fibroblast subpopulations under perturbed conditions in skin and oral mucosa. (**A**) Left: skin wound healing typically results in scar formation and involves multiple fibroblast subsets. During the inflammatory phase, *APOD*^+^/*APOE*^+^ fibroblasts express *CCL19*, which can recruit inflammatory leukocytes. *PRG4*^+^ proinflammatory fibroblasts also emerge early during the inflammatory phase in the reticular dermis and are later replaced by *COL25A1*^+^ fibroblasts found in the scar. During the proliferative phase of healing, *CRABP1*^+^ fibroblasts are localized to the upper part of the wound, while *DPP4*^+^ mechanofibrotic fibroblasts occupy the deep wound region. *MKI67*^+^ fibroblasts also transition into *COL11A1*^+^/*POSTN*^+^ mesenchymal fibroblasts during the remodeling phase. The accumulation of collagen-rich mechanofibrotic fibroblasts in the later phase of wound healing likely contributes to scar formation in skin. These fibroblasts can be directly targeted by using genetically modified murine models that label *PRRX1* or *EN1* lineage–positive fibroblasts. Right: oral wound healing rarely forms a scar and involves a specialized *Prrx1^hi^* fibroblast progenitor population that is highly proliferative upon injury. This subset differentiates into *SCA1*^+^ fibroblasts that rapidly express *CXCL1* and *CCL2* to attract innate immune cells and facilitate swift resolution of inflammation. Other oral fibroblast subsets, such as *PI16*^+^ cells, remain uncharacterized in the oral wound-healing literature. (**B**) Left: fibroblast subsets characterized in the skin of patients with atopic dermatitis and psoriasis. In atopic dermatitis, *COL6A5*^+^ *COL18A1*^+^ fibroblasts have been reported to express *CCL19*, which can recruit *CCR7*^+^ dendritic cells, and *CCL2*, which can recruit Th2 and Th22 cells. *COL6A5*^+^ fibroblasts are also the source of *CCL11* and *CCL26*, which promote eosinophil recruitment, and *CCL27*, which recruits memory T cells. In psoriasis, *WNT5A*^+^ fibroblasts have been reported to express *IL24*, which may communicate with spinous keratinocytes. *SFRP2*^+^ fibroblasts contribute to psoriatic inflammation by recruiting *CCR7*^+^ dendritic cells and *CCR2*^+^ myeloid cells. They also recruit *CXCR4*^+^ *CD8*^+^ T cells, which release *IL17* to promote type 1 and 17 inflammation. Right: oral fibroblast subsets identified in periodontitis. These include *ICAM1*^+^ and *COL14A1*^+^ fibroblasts that express *CXCL1* and/or *CCL2* for myeloid cell recruitment. *IL36R*^+^ and *RAC2*^+^ *LCP1*^+^ fibroblasts have been described and express *CXCL2* and *CXCL8*, respectively, for neutrophilic inflammation. *RAC2*^+^ *LCP1*^+^ fibroblasts also express *CXCL10*, which may interact with *CXCR3*^+^ macrophages and T cells. Human fibroblast subsets are illustrated unless indicated as follows: ^*^Fibroblast subset reported from human and murine models. ^†^Fibroblast subset reported in murine models only.

In a recent study, Liu et al. (2025) employed scRNA-seq and spatial transcriptomic approaches to examine human skin wound repair over 1-, 7, and 30-d time points. The study identified 4 major fibroblast clusters, each designated as a mesenchymal (*POSTN^+^*), papillary (*ELN^+^LEPR^+^*), inflammatory (*C3^+^*), and proliferating fibroblast subset (*MKI67^+^*). The proliferative fibroblasts emerged mainly on day 7 and were then replaced by *COL11A1^+^*/*POSTN^+^* mesenchymal fibroblasts that expressed collagen-associated genes by day 30. In contrast, *APOD+*/*APOE+* proinflammatory fibroblasts expressed inflammation-related genes such as *C3* and *CCL19* and declined in numbers from day 7 to 30. The increasing composition of the *COL11A1^+^*/*POSTN^+^* fibroblast subset in a later stage of wound healing implicates a role in scar formation since elevated levels of *POSTN* and *COL11A1* are found in human fibrotic diseases (Deng et al. 2021; Ma et al. 2024). When compared with the skin wound-healing literature, scRNA-seq studies that examine oral wounds across multiple time points are scarce, although mechanistic studies that examine select fibroblast subpopulations are available.

### Functional In Vivo Oral and Skin Wound-Healing Studies

scRNA-seq studies have identified distinct fibroblast subsets during wound healing, but their biological relevance is underexplored. Our group reported that postnatal fibroblasts with active *Prrx1* enhancer element (Prx1^enh+^) are abundant in the anterior hard palate but minimal in skin (Ko et al. 2023). These fibroblasts become highly proliferative upon oral wound induction and differentiate toward *SCA1*^+^ fibroblasts to expedite healing via CCL2 expression and macrophage recruitment (Fig. 3A). Moreover, genetic deletion of *Ikbkb* in *Prrx1* lineage–positive oral fibroblasts suppresses their inflammatory function and delays wound healing in the palate (Chen et al. 2024), highlighting the role of immunomodulatory fibroblasts for appropriate oral wound healing. In contrast, skin wound-healing literature has focused on inhibiting profibrotic fibroblast subpopulations to reduce scar formation. For instance, genetic ablation of *Prrx1* lineage–positive ventral skin fibroblasts in mice can abrogate scarring in skin wounds (Leavitt et al. 2020). In addition, *DPP4*^+^ skin fibroblasts are labeled by *Engrailed-1^+^* lineage and possess an intrinsic ability to form scar in skin injury (Rinkevich et al. 2015). *Engrailed-1* expression in skin fibroblasts is also induced by applying tension to injured wounds via mechanotransduction pathways (Mascharak et al. 2021). These mechanistic studies offer valuable insights into developing future therapeutic strategies that precisely target select fibroblast subsets to achieve a more regenerative healing outcome.

## Fibroblast Subpopulations in Chronic Inflammatory Conditions

### Periodontal Disease

Periodontitis is a prevalent chronic inflammatory disease characterized by persistent connective tissue inflammation and progressive destruction of tooth-supporting structures. There exists abundant scRNA-seq studies that investigate the cellular and transcriptional dynamics in human periodontitis (Caetano et al. 2021; Williams et al. 2021; Caetano et al. 2023). These studies collectively point to an inflammatory gingival fibroblast subset expressing CCL- or CXCL- cytokines as a prominent feature when comparing periodontitis with healthy groups (Fig. 3B). Williams et al. (2021) demonstrated that the fibroblast subset marked by *APOD* and *COL11A1* is more abundant in periodontitis as compared with a healthy group and is predicted to promote the recruitment of *CXCR1^+^*/*CXCR2^+^* neutrophils by expressing *CXCL1*, *CXCL2*, *CXCL5*, and *CXCL8*. Cell-cell ligand-receptor analysis also implicated interactions between fibroblasts that express *CCL2*, *CXCL12*, *CX3CL1*, and *CCL19* and leukocytes that express cognate receptor genes such as *CX3CR1^+^CCR2^+^* monocytes/macrophages and *CCR7^+^CXCR4^+^* lymphocytes in periodontitis. Caetano et al. (2023) utilized spatial transcriptomics and identified an inflammatory fibroblast subpopulation that expresses *CXCL8* and *CXCL10* to be localized in proximity to leukocytic infiltration that included neutrophils, *CXCR3*^+^ macrophages, and T cells. While these human scRNA-seq datasets point to a potential role of inflammatory fibroblast subsets in periodontitis progression, they represent a snapshot of an established lesion and thus require a closer examination across varying time courses using animal models.

### Murine Studies on Fibroblasts in Periodontitis

Several studies have investigated the role of fibroblasts using murine models of periodontitis. Kondo et al. (2022) demonstrated that *Col14a1^+^* fibroblasts rapidly upregulate *Ctsk* to initiate connective tissue degradation as early as 1 d after ligature placement. A follow-up study demonstrated that *Col14a1^+^* fibroblasts highly express *Cxcl1* and *Cxcl12* for recruiting neutrophils as well as *Il6* and *Cxcl10* for activation of *Il17a*/*f-*expressing innate lymphoid cells (Kondo et al. 2023). Another study reported that IL36R^+^-responsive gingival fibroblasts express *Cxcl1* and *Cxcl12* to recruit destructive neutrophils (Liu et al. 2024). The detrimental impact of neutrophils is also supported by the functional studies that inhibit their inflammatory functions (Kim et al. 2023; Qiu et al. 2024), which may be driven by gingival fibroblasts. By leveraging the existing human scRNA-seq database (Caetano et al. 2021; Williams et al. 2021), our group recently reported that ICAM-1 (intercellular adhesion molecule 1) is a reliable surface marker that identifies inflammatory fibroblasts when combined with lineage-negative and pan-fibroblast markers in human and mouse models (Kim et al. 2024). Genetic inhibition of inflammatory NF-κB activity in *Prrx1* lineage–positive fibroblasts, which labeled nearly 80% of ICAM-1^+^ fibroblasts, paradoxically potentiated bone loss and caused excessive neutrophilic infiltration in ligature-induced periodontitis. This was partly explained by the failure to clear neutrophilic bodies by macrophages whose recruitment depended on CCL2 expression by the ICAM-1^+^ gingival fibroblasts.

### Chronic Inflammatory Diseases of the Skin

Similar to oral mucosa, the skin barrier is susceptible to chronic inflammation (Fig. 3B). Atopic dermatitis (AD) is a prevalent chronic skin disease characterized by overt type 2 inflammation and allergic pruritus. scRNA-seq studies on human atopic skin have largely validated dysregulated epithelial barriers and inflammatory milieu but also revealed unexpected changes in dermal fibroblasts. Reynolds et al. (2021) reported that the number of fibroblast subsets that expresses *CXCL12* and *CCL19* is significantly higher in AD lesions when compared with healthy skin. Consistent with this, He et al. (2020) demonstrated that human atopic lesions had increased numbers of a *COL6A5^+^COL18A1^+^* fibroblast subpopulation that also expressed *CCL19* and *CCL2* when compared with nonlesional skin. Spatial transcriptomic analysis revealed that this fibroblast subset was found near the inflammatory foci, which contained *CCR7*^+^ dendritic cells and an increased frequency of type 2 and 22 T-helper cells in AD skin (Mitamura et al. 2023).

Psoriasis is another common chronic skin disease characterized by thick scaly plaques and is biased toward type 1 and 17 inflammation (Greb et al. 2016). scRNA-seq studies on psoriasis identified a proinflammatory fibroblast population marked by upregulated expression of *CCL19*, *TNFSF13B*, and *CXCL12* (Hughes et al. 2020). In another study of psoriasis, Ma et al. (2023) reported that *SFRP2^+^* fibroblast subpopulation transitions from a profibrotic to proinflammatory state and becomes a major producer of cytokines such as *CCL13*, *CCL19*, and *CXCL12*. These cytokines were predicted to interact with recipient cells such as *CCR2^+^* myeloid cells, *CCR7^+^* dendritic cells, and *CXCR4^+^* cytotoxic T cells that express *IL17A*/*F*. In a study that directly compared AD and psoriasis by scRNA-seq, the immune signature in the fibroblasts from AD was more prominent than that of psoriasis such that there was an increased expression of *COL6A5*, *CCL27*, and *CCL26* with the latter 2 being chemoattractant for memory T cells and eosinophils (Zhang et al. 2023). Similarly, a recent study identified a *WNT5A^+^IL24^+^* fibroblast population to be uniquely present in psoriatic skin but not in healthy or AD skin and that this fibroblast subset disappeared upon disease remission (Francis et al. 2024).

### Functional Studies on Fibroblasts in Skin Inflammatory Diseases

In a murine AD study using epicutaneous ovalbumin sensitization, fibroblast subsets exhibited an inflammatory phenotype marked by increased expression of *Ccl2*, *Ccl7*, *Ccl8*, and *Cxcl5* as compared with control groups (Leyva-Castillo et al. 2022). Similarly, in a murine psoriasis model that used topical imiquimod, fibroblast subpopulations displayed enriched expression of *Ccl2*, *Cxcl12*, and *Cxcl14*, which was minimal in control groups (Guo et al. 2024). These murine studies show that fibroblast subpopulations are an important effector cell type that amplifies inflammatory sequalae in chronic skin diseases. Recent studies that target dermal fibroblasts by genetic deletion of *Ikbkb* demonstrated that perturbation of fibroblasts alone was sufficient to induce AD-like symptoms in mice (Nunomura et al. 2019; Ko et al. 2022). This was due to the upregulation of eosinophil chemoattractant *Ccl11* in a *Col6a5^+^* fibroblast subset, which caused excessive eosinophilia and amplification of a type 2 immune response in the skin (Ko et al. 2022). Table 2 summarizes murine and human scRNA-seq studies that include fibroblast analyses on chronic inflammatory conditions in skin and oral mucosa.

**Table 2.** Single-Cell RNA Sequencing Studies of Fibroblasts for Perturbed Conditions in Skin and Oral Mucosa.

Reference: Species and Disease Model	FB Subsets Reported	Inferred Function/Identity	Experimental Validation in FB: Database Identifier
**Skin wound-healing studies**			
Foster et al. (2021) Mouse Excisional skin wound healing (POD 2, 7, 14)	Cluster 1: *Col3a1*+, *En1*+, *Ccl2*+, *Dpp4*+ Cluster 2: *Cxcr5*+, *Cxcr2*+, *Il1a*+, *Fn1*+ Cluster 3: *Ccl6*+, *Il1b*+, *Osm*+, *Itga4*+, *Il6st*+ Cluster 4: 4a: *Stat3*+, *Lef1*+, *Runx3*+, *Cxcr6*+ 4b: *Itgae*+, *Mmp25*+, *Ccr4*+, *Cxcr4*+ 4c: *Ccr6*+	Cluster 1: mechano-fibrotic response Cluster 2: immune activation and response Cluster 3: tissue remodeling Cluster 4: proliferation	scATAC-seq confirmed cluster 1 as a mechanofibrotic subset that showed increased chromatin accessibility proximal to key fibrosis-related genes • GSE178758
Almet et. al. (2025) Mouse Skin: small wound <1 cm (POD 2,7); large wound >1 cm (POD 12, 14, 18)	FIB-I: *Crabp1^+^*, *Col7a1^+^*, FIB-II: *Col14a1^+^*, *Mgp^+^*, FIB-III: *Pcolce2^+^*, *Ndufa4/2^+^*, FIB-IV: *Plac8^+^*, *Ptx3^+^*, *Prg4^+^* FIB-V: *Tyrobp^+^*, *Lyz2^+^*, FIB-VI: *Col5a3^+^*, *Prss23* FIB-VII: *Igfbp2^+^*, *Megf6^+^*, *Col25a1^+^*, DP: *Igfbp3^+^*, *Bmp4^+^* FIB-VIII: *Coch^+^*, *Dkk2^+^* FIB-IX: *Lgals7^+^*, *S100a14^+^* FIB-X: *Nr2f2^+^*, *Cldn1^+^*	FIB-IV: *Prg4^+^* early wound responder in reticular layer FIB-VII: *Col25a1^+^* late wound FB detected in lower scar region FIB-I, II, III, V, VI, VIII, IX, X, and DP: not specified	Immunofluorescence confirmed *Prg4^+^* FBs in lower wound region in early wound healing, and *Col25a1^+^* FBs emerged after epithelial wound closure. • GSE153596 • GSE142721 • GSE113854 • GSE108677 • GSE141814
Leavitt et al. (2020) Mouse 6-mm ventral skin wound (POD 14)	Cluster 0: *Col1a1^+^*, *Cdh4^+^* Cluster 1: *Lpl^+^*, *Gpx3^+^* Cluster 2: *Megf6^+^* Cluster 3: *Mcoln2^+^* Cluster 4: *Galnt15^+^* Cluster 5: *Prg4^+^*	Cluster 4: *Prrx1^-^* FB Cluster 5: *Prg4^+^* FB comprised primarily *Prrx1^+^* FB, implicated in fibrosis, collagen production, and FAK signaling Cluster 0 to 3: not specified	Genetic ablation of *Prrx1* lineage–positive FB abrogates scarring in skin wounds • GSE159345
Liu et al. (2025) Human 4-mm full-thickness wounds in upper buttock (POD 1, 7, 30)	FB: *ELN^+^*, *SFRP4^+^* FB: *SFRP1^+^*, *CRABP1^+^* Subgroups: FB-I: *POSTN^+^*, *COL4A1^+^*, *ADAM12^+^* FB-I: *POSTN^+^*, *MMP11^+^*, *ADAM12^+^* FB-I: *SFRP4^+^*, *COMP^+^* FB-I: *POSTN^+^*, *COL11A1^+^* FB-II: *APOE^+^*, *CCL19^+^* FB-II: *APOD^+^*, *ITM2A^+^* FB-III: *ELN^+^*, *LEPR^+^* FB-prolif: *MKI167^+^*	FB-I: mesenchymal FB FB-II: proinflammatory FB FB-III: papillary FB FB-prolif: proliferating FBs express *HGF*, *FGF2*, and *TGFβ1* in proliferative phase	RNA FISH confirmed that *ADAM12^+^* mesenchymal FBs populate wound at late stage of wound healing (POD 30) • GSE241132 • GSE265972
**Chronic inflammation at the oral mucosal barrier**			
Caetano et al. (2021) Human Inflamed gingiva, periodontitis	S0: *PDGFRA^+^*, *WNT5A^+^*, *IGF1^+^*, *POSTN^+^* S2: *GREM1^+^*, *SERP1^+^*, *APCDD1^+^*, *DKK3^+^* S4: *OSR2^+^*, *FGFR1^+^*, *SOX4^+^*, *TBX3^+^* S5: *ILR1^+^*, *IFNGR1^+^*, *TNFRS11B^+^* S6: *CD74^+^*, *AREG^+^*	S0: tissue repair S2: negative regulation of WNT S4: collagen IV, development S5: IFN-γ signaling and osteoclast activity S6: IFN-γ signaling and T-cell activation	NA • GSE152042
Williams et al. (2021) Human Inflamed gingiva, periodontitis	P.Fib 1.1: *CXCL13^+^*, *CXCL2^+^*, *CXC1^+^* P.Fib 1.2: *APCDD1^+^*, *IGFBP2^+^*, *MRPS6^+^* P.Fib 1.3: *APOD^+^*, *GSN^+^*, *CFD^+^* P.Fib 1.4: *TIMP3^+^*, *ASPN^+^*, *COL11A1^+^*	Recruitment of neutrophils and other leukocytes	NA • GSE164241
Caetano et al. (2023) Human Inflamed gingiva, periodontitis	Fb1: Reticular FB Fb2: *GREM1^+^*, *SFRP1^+^* Fb3: Reticular FB Fb4: Immune-associated Fb5: *RAC2^+^*, *LCP1^+^*, *TNFRS11B^+^*	Fb2: epithelial maintenance through *RSPO1*, *CLEC11A* and *FLRT2* Fb5: cytokine production (*CXCL8*, *CXCL10*) Fb1, 3, 4: not specified	Immunostaining validated Fb5 to be localized in B cell–enriched inflammatory region • GSE206621
Qiu et al. (2024) Human Inflamed gingiva, periodontitis	*MIF^+^* FBs	Recruit CD74^+^/CXCR4^+^ neutrophils to promote production of neutrophil extracellular traps	NA • Available upon request
Kim et al. (2024) Human Inflamed gingiva, periodontitis	FB 0: *ICAM1^+^* FB 1-5: not specified	ICAM1^+^ FB: immunomodulatory function expressing *CXCL1* and *CCL2*	Enriched expression of *CCL2* and *CXCL1* in *ICAM1^+^* vs. *ICAM1^-^* FB confirmed by quantitative polymerase chain reaction and immunostain • GSE164241 • GSE217720 • GSE152042
Kondo et al. (2022) Mouse Ligature-induced periodontitis gingiva (POD 1, 3, 5, 7)	Fib1: not specified Fib2: *Col14a1^+^*	Fib2: expresses *Ctsk* to promote connective tissue degradation and upregulated proinflammatory transcripts	Immunofluorescence demonstrated *Col14a1^+^* FBs in papillary layer of gingival connective tissue • GSE201108 • GSE201109
Kondo et al. (2023) Mouse Ligature-induced periodontitis gingiva (POD 0, 1, 4, 7)	AG: *Col14a1^+^* KT: not specified MF: *Acta2^+^*	AG: expresses *Cxcl1* and *Cxcl12* for neutrophil recruitment KT: connective tissue remodeling MF: responds to pathogens via *Tlr2* and *Tlr4*	Immunohistochemistry showed colocalization of *Col14a1* and *Cxcl12* • GSE201108 • GSE201109 • GSE228635
**Cutaneous chronic inflammatory conditions**			
Hughes et al. (2020) Human Multiple skin conditions from multiple locations	Cluster 0: *PI16^+^*, *ITIH5^+^* Cluster 1: *POSTN^+^*, *MMP11^+^* Cluster 2 and 8: *LTBP4^+^*, *IGFBP5^+^*, *TCF4^+^* Cluster 3: *COL11A1^+^*, *DPEP1^+^*, *RBP4^+^* Cluster 4: *CCL19^+^*, *CXCL12^+^* Cluster 5: *SFRP2^+^*, *PRSS23^+^*, *IL6^+^* Cluster 7: *SPOCK1^+^*, *CRLF1^+^*, *COMP^+^*	0: enriched in GA 1: enriched in leprosy 2 and 8: enriched in normal skin 3: connective tissue remodeling 4: enriched in leprosy, proinflammatory FBs 5: enriched in leprosy, proinflammatory FBs 7: enriched in granuloma annulare	NA • GSE150672
Reynolds et al. (2021) Human Healthy, atopic dermatitis and psoriasis from multiple locations	FB1: not specified FB2: *COL1A1^+^*, *COL1A2^+^*, *COL6A1^+^* FB3: Not specified	FB1 and FB3: not specified FB2: chemotactic function via *CXCL12* and *CCL19*, enriched in atopic dermatitis and psoriasis	NA • https://developmental.cellatlas.io/diseased-skin
Mitamura et al. (2023) Human Atopic dermatitis from antecubital fossa skin	FB-1: *MFAP5^+^* FB-2.1: *COL18A1^+^* FB-2.2: *APOE^+^* FB-2.3: *SFRP1^+^*	FB-1 and FB-2.2: upregulate *FGF7* gene FB-2.1: expresses *CCL19* and *IL32*, communicates with leukocytes FB-2.3: not specified	Immunofluorescence showed increased expression of *COL18A1* in lesional skin of atopic dermatitis • GSE153760 • GSE197023
He et al. (2020) Human Atopic dermatitis from extremities	1: *APOE^+^*, *ABCA6^+^* 2: *FBN1^+^*, *MFAP5^+^* 3: *COL11A1^+^*, *LAMC3^+^* 4: *COL6A5^+^*, *COL18A1^+^*	1 and 2: homeostasis and lipid transport 3: resemble fibrocartilage/myofibroblast 4: express inflammatory *CCL2*, *CCL19*, and *IL32*	Immunofluorescence showed increased expression of *COL6A5* and *MFAP5* in atopic dermatitis • GSE147424
Zhang et al. (2023) Human Atopic dermatitis, psoriasis from multiple locations	AD: *POSTN^+^*, *IFITM2^+^*, *IFITM3^+^* PS: *APOE^+^*, *THY1^+^*	AD: type 2 inflammation	NA • NA
Francis et al. (2024) Human Psoriasis (IL-23 blockade treatment) from lower back/buttocks	1: *CCN5^+^*, *PI16^+^* 2: *COMP^+^* 3: *COMP^+^* 4: *COMP^+^* 5: *WNT5A^+^*, *IL24^+^* 6: *APOE^+^*, *CXCL3^+^* 7: *APOE^+^*, *CCL19^+^* 8: *APOE^+^*, *C7^+^* 9: *COL11A1^+^*, *DPEP1^+^* 10: *COCH^+^*, *ASPN^+^* 11: *ANGPTL7^+^*	5: *WNT5A^+^* *IL24^+^* proinflammatory FB communicates with spinous keratinocytes 7: inflammatory FB expresses CCL19 9: inflammatory FBs 1 to 4, 6, 8, 10, 11: not specified	Immunofluorescence showed that *WNT5A^+^* cells are detected in lesional psoriasis skin and the number of *WNT5A^+^* cells reduces after IL23 blockade treatment • GSE228421 • GSE202011 • GSE222840 • GSE114729
Ko et al. (2022) Mouse Spontaneous atopic dermatitis model from ventral skin	WT: *Palld^+^*, *Pi16^+^* cKO: *Col6a5^+^*, *Coch^+^* Mixed WT/cKO: not specified	WT: microfilament reorganization and regulation of neuropathic pain cKO: upregulate *Ccl11*, *Cxcl12*, and *Ccl7* to promote eosinophilia and type 2 immunity	Immunofluorescence showed increased *Ccl11^+^* FB and eosinophilia in *Prx1Cre^+^Ikbkb*^f/f^ experimental mice • GSE172226
Leyva-Castillo et al. (2022) Mouse Ovalbumin sensitization atopic dermatitis model from dorsal skin	1: Col-I producing Fb: *Col1a1^+^*, *Col1a2^+^* 2: Col-VIII/IX producing Fb: *Col8a1^+^*, *Col8a2^+^*, *Col9a1^+^*, *Col9a2^+^* 3: dermal papilla Fb: *Sst^+^*, *Hhip^+^*, *Sox2^+^* 4: myofibroblast: *Myod^+^*, *Lypd^+^* 5: lipofibroblast: *Mfap^+^*, *Cygb*, *Apod^+^*	1: inflammatory FB expressing *Ccl8*, *Col5a2*, *Cxcl5*, *Il33* 2: inflammatory FB expressing *Ccl2*, *Ccl7*, *Fn1* 3: matrix production 4: not specified 5: inflammatory FB expressing *Ccl8*, *Col5a2*, and *Cxcl5*	NA • GSE194254
Guo et al. (2024) Mouse Imiquimod-induced psoriasis model from ear and dorsal skin	Angiogenic Fib: *Gpx3^+^*, *Plac8^+^*, *Vegfd* ^+^ iFib1: *Cxcl12^+^*, *Cxcl14^+^*, *Prg4^+^* iFib2: *Ccl2^+^*, *Ctsk^+^*, *Coch^+^* Mitotic Fib: *Hmgb2^+^*, *Pclaf^+^*,*Top2a^+^* Normal Fib: *Wif1^+^*, *Enpp2^+^*, *Serpine2^+^* Pericytes like: *Acta2^+^*, *Tagln^+^*, *Myh11^+^* Spp1 Fib: *Apod^+^*, *Cp^+^*	Angiogenic Fib, Mitotic Fib, Pericytes, *Spp1* Fib: not specified iFib1 and iFib2: inflammatory FB, promotes epithelial-mesenchymal transition	NA • GSE165021

FB, fibroblast; NA, not available; POD, postoperative day.

## Concluding Remarks and Future Perspectives

Fibroblasts constitute heterogeneous subpopulations, each equipped with a specialized gene expression profile and spatial distribution in skin and oral mucosa. Other than extracellular matrix–associated genes, these fibroblast subsets differentially express transcripts that are implicated in inflammation and distinct molecular pathways. A major limitation in interpreting individual scRNA-seq studies is the inconsistent cell nomenclature and varying number of cell identities. This likely stems from a combination of technical variations, such as cell dissociation protocols and/or computational workflow, which can vary widely among studies. To address this, an integrative approach to categorize fibroblast subsets has been performed for skin fibroblasts (Ascensión et al. 2021; Almet et al. 2025), which consistently identified *DPP4*^+^ fibrosis-associated fibroblasts, *C7*^+^ immunomodulatory fibroblasts, and *CRABP1*^+^ wound-remodeling fibroblasts. Another limitation is that fibroblast-specific genes found in mouse models may not be found in human fibroblasts, such as *Lrig1*, *Dlk1*, and *Ly6a.* In a recent study, Ascensión and Izeta (2024) performed comparative analysis of mouse and human skin fibroblasts and identified multiple conserved subsets, including those expressing *CXCL12* and *IL6*.

An integrated scRNA-seq atlas of human oral and craniofacial tissues is available (Fernandes Matuck et al. 2024). However, a detailed fibroblast-centric analysis across oral mucosal sites is lacking and remains a promising future direction. In the oral cavity, different anatomic sites are under various functional demands, and site-specific disease susceptibility is a well-known phenomenon. Similar fibroblast features implicated in chemotaxis and complement activation are found in healthy gingiva and buccal mucosa (Williams et al. 2021). This may be explained by the shared neural crest developmental origin and/or exposure to oral microbiota. Yet, it is worth noting that fibroblasts in these anatomic sites have not been compared under perturbed conditions (i.e., wounding), which may reveal divergent molecular pathways. An integrative oral fibroblast atlas may also identify stable and intrinsic markers for distinct oral fibroblast subset that must precede development of new mouse models for mechanistic studies in the future.

While informative, scRNA-seq analysis alone cannot resolve the spatial context of cells in a given niche. Spatial transcriptomics is a rapidly evolving methodology that can unravel gene expression patterns within complex tissues, and its application for examining oral structures is continually expanding (Caetano and Sharpe 2024; Haller et al. 2024). In the skin biology field, this technology has been employed for human wound healing and chronic inflammatory diseases conditions (Ganier et al. 2024; Steele et al. 2024; Liu et al. 2025). As the detection voxel size improves down to 2 µm (Visium HD) and the single-cell resolution becomes capable of detecting up to 5,000 transcripts (Xenium), we anticipate that future studies will provide a spatial context to oral fibroblast identity and function at an unprecedented resolution. A comprehensive analysis of various oral tissues in health and disease will be the crucial next step toward understanding remarkable fibroblast heterogeneity in the oral cavity, identifying functionally relevant fibroblast subsets, and developing precision therapy targeting distinct subpopulations.

## Author Contributions

K. Prasongyuenyong, K.I. Ko, contributed to conception and design, data acquisition, analysis, and interpretation, drafted and critically revised manuscript; W.S. Kim, contributed to conception and design, data acquisition, analysis, and interpretation, drafted and critically revised manuscript; Z. Chen, contributed to conception and design, drafted the manuscript. All authors gave final approval and agreed to be accountable for all aspects of the work.
